# Symptomatic pulmonary cement embolism following PMMA vertebroplasty: Clinical presentation and management

**DOI:** 10.1016/j.rmcr.2025.102325

**Published:** 2025-11-05

**Authors:** Haytham Najjar, Hind Neiroukh, Layth J.M. Saada, Mohamad Thalji, Kataeb Doudin

**Affiliations:** a Internal Medicine Department, Indiana University School of Medicine-muncie, Indiana, USA; b Emergency Department, Al-Mezan Speciality Hospital, Hebron, West bank, Palestine; c Al-Quds University, Jerusalem, Palestine; d Surgery Department, Al-Mezan Speciality Hospital, Hebron, West bank, Palestine; e Internal Medicine Department, Al-Mezan Speciality Hospital, Hebron, West bank, Palestine

**Keywords:** Bone cement, Pulmonary cement embolism, Vertebroplasty, Polymethylmethacrylate (PMMA), Postoperative dyspnea, Non-thrombotic pulmonary embolism

## Abstract

**Background:**

Vertebroplasty, a minimally invasive technique frequently used for spine stabilization and vertebral compression fractures, uses polymethylmethacrylate (PMMA) bone cement. Although usually benign, pulmonary cement embolism (PCE) is an uncommon but potentially dangerous consequence that arises when bone cement enters the pulmonary circulation through the vertebral venous system. Although the majority of PCE cases are underdiagnosed and asymptomatic, the incidence varies between 2.1 % and 26 % of vertebroplasty surgeries. To prevent incorrect diagnoses and needless treatments, early detection is essential.

**Case presentation:**

We present the case of a 71-year-old woman with a history of type 2 diabetes mellitus and hypertension. She underwent laminectomy at the L3/4 and L4/5 levels in addition to PMMA cement-augmented intrapedicular fixation because of her severe spinal stenosis, hypertrophic facet joints, and instability. Three days following surgery, the patient had severe respiratory symptoms, including tachypnea, tachycardia, hypoxemia, and dyspnea. Additional oxygen was required due to oxygen desaturation. Non-contrast chest CT confirmed the presence of multiple bilateral high-density branching opacities in the pulmonary arteries that were compatible with PMMA emboli.

**Conclusion:**

This example emphasizes how difficult it can be to diagnose symptomatic PCE after spinal instrumentation and how crucial it is to keep a high level of suspicion when patients arrive with unexplained respiratory symptoms after vertebroplasty. The preferred diagnostic method remains non-contrast chest CT, and the majority of symptomatic individuals can be conservatively treated with extra oxygen and anticoagulation when necessary.

## 1 Introduction

Percutaneous vertebroplasty and kyphoplasty have become increasingly popular minimally invasive procedures for the treatment of spinal compression fractures, particularly those caused by osteoporosis, trauma, or neoplastic involvement. Since Galibert et al. first described vertebroplasty in 1987, it has demonstrated substantial efficacy in reducing pain, stabilizing vertebral architecture, and improving functional outcomes through the targeted injection of polymethylmethacrylate (PMMA) cement under fluoroscopic guidance [1].

While vertebral augmentation treatments are generally regarded as safe, there are certain risks associated with them. Pulmonary cement embolism (PCE) is an uncommon adverse event that is potentially dangerous among the known consequences. PCE occurs when PMMA cement inadvertently penetrates the venous system during surgery and subsequently reaches the pulmonary artery. Despite the fact that PCE is often asymptomatic and unintentionally discovered, it can occasionally present with severe cardiopulmonary compromise and therefore requires immediate identification and treatment [2,3].

PCE manifests clinically in a variety of ways, from subtle radiologic abnormalities to severe respiratory distress, and the published prevalence is inconsistent. Venous anatomy, vertebral level treated, and cement volume and viscosity are among the risk factors. Diagnostic imaging necessitates high-resolution imaging, particularly non-contrast chest CT, which facilitates the identification of radiodense embolic material. The management approach is contingent upon the severity of the symptoms, which may involve observation, anticoagulation, or, in life-threatening situations, surgery [4,5].

We present a case of symptomatic PCE following vertebroplasty, thereby highlighting the challenges associated with diagnosing and treating an underdiagnosed complication. The objective of this paper is to enhance clinical knowledge and contribute to the existing body of knowledge concerning the safe application of vertebral augmentation techniques.

## 2 Case Presentation

A 71-year-old female with a medical history of hypertension and type 2 diabetes mellitus, as well as a prior surgical history of cholecystectomy, presented to our surgical facility with progressively debilitating lower back pain that had been present for several years and was radiating to the left lower limb. Her symptoms included impaired sphincter control, motor paralysis, and numbness. Decompression and stabilization were recommended in response to the severe lumbar spinal stenosis, facet joint hypertrophy, and instability that were identified by magnetic resonance imaging (MRI) of the lumbosacral spine.

The patient was clinically stable prior to surgery, with normal vital signs, no fever, and no respiratory complaints. The patient's cardiorespiratory examination demonstrated bilaterally clear breath sounds without wheezes, crackles, or any other anomalous findings. She subsequently underwent laminectomy and polymethylmethacrylate (PMMA) cement-augmented intrapedicular fixation at the L3/4 and L4/5 levels.

**Fig. 1:**
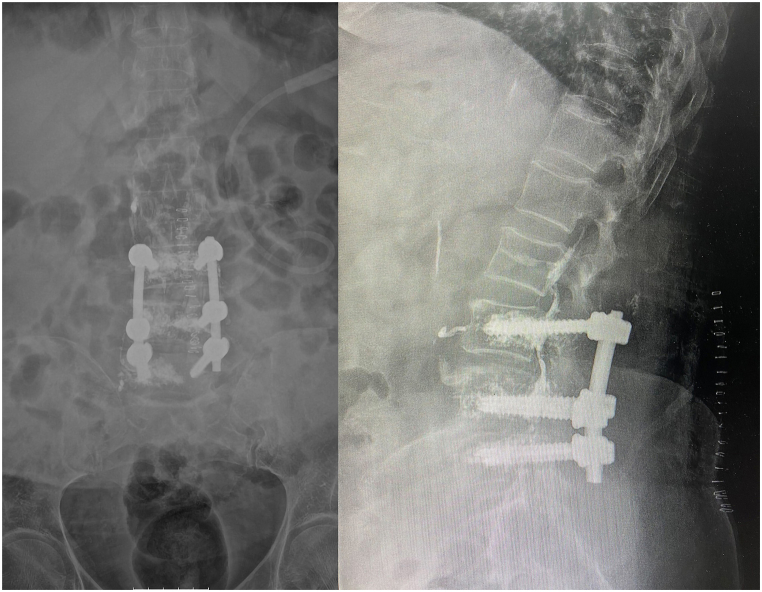
Postoperative anteroposterior and lateral lumbosacral spine radiographs demonstrating cement-augmented pedicle screw fixation at the L3/4 and L4/5 levels.

The patient experienced moderate, transient hypotension and oxygen desaturation on the first postoperative day, with a peak D-dimer level of 4925 ng/mL. Further imaging was postponed as she maintained clinical stability and improved with intravenous fluid resuscitation. An X-ray of the lumbosacral spine (Fig. 1) was also obtained as part of the standard postoperative evaluation.

Nevertheless, on the third postoperative day, she experienced new-onset shortness of breath, tachypnea (respiratory rate: 23 breaths/min), tachycardia (heart rate: 105 beats/min), and hypoxemia (SpO_2_ 90 % on 4–5 L/min via nasal cannula). Her blood pressure was 120/60 mmHg, and her temperature was 36.9 °C. The right middle and lower lung zones exhibited a reduction in breath sounds and crackles during auscultation. Subsequently, she was transferred to the intensive care unit for additional management. A leukocytosis (WBC 14.5 × 10^9^/L), anemia (hemoglobin: 10.6 g/dL), and a significantly elevated C-reactive protein (CRP: 187 mg/L) were observed in the laboratory investigations. Troponin was within normal limits. On the third day, the D-dimer level had decreased to 407 ng/mL. The lactate level was within an acceptable range (0.4 mmol/L), and the arterial blood gas analysis revealed respiratory alkalosis (pH 7.49, pCO_2_ 29.7 mmHg, HCO_3_^−^ 23.1 mmol/L). The remaining laboratory parameters were unremarkable.

Chest computed tomography pulmonary angiography (CTPA) ruled out thromboembolic pulmonary embolism. However, it demonstrated extensive bilateral ground-glass opacities (right-sided predominance), which are indicative of ARDS versus atypical chest infection. Additionally, the CTPA revealed moderate right and mild left pleural effusions with atelectasis, as well as multiple linear calcifications that suggest a previous infection. The patient's transthoracic echocardiography revealed that their left ventricular systolic function was preserved at 65 %, they had moderate pulmonary hypertension (RVSP 48 mmHg), a small IVC with inspiratory collapse, and there were no indications of significant right heart strain or acute pulmonary embolism.

Furthermore, the patient's respiratory symptoms persisted despite incentive spirometry and broad-spectrum antibiotic therapy. All cultures from blood, urine, sputum, and nasal/rectal samples were negative, and procalcitonin remained within normal ranges. The CT findings on postoperative day 3 were consistent with the follow-up chest X-ray on postoperative day 5, which revealed an increase in right-sided ground glass opacities and pleural effusion (Fig. 2a). These findings were not present on the preoperative chest radiograph (Fig. 2b).

**Fig. 2a:**
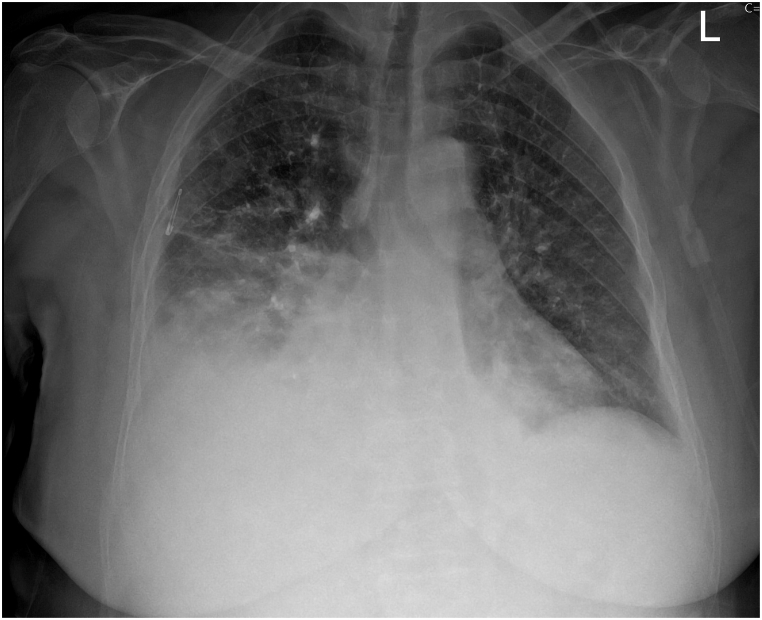
Chest radiograph on postoperative day 5 showing increased right-sided opacities and pleural effusion, with new infiltrates consistent with evolving lung pathology.

**Fig. 2b:**
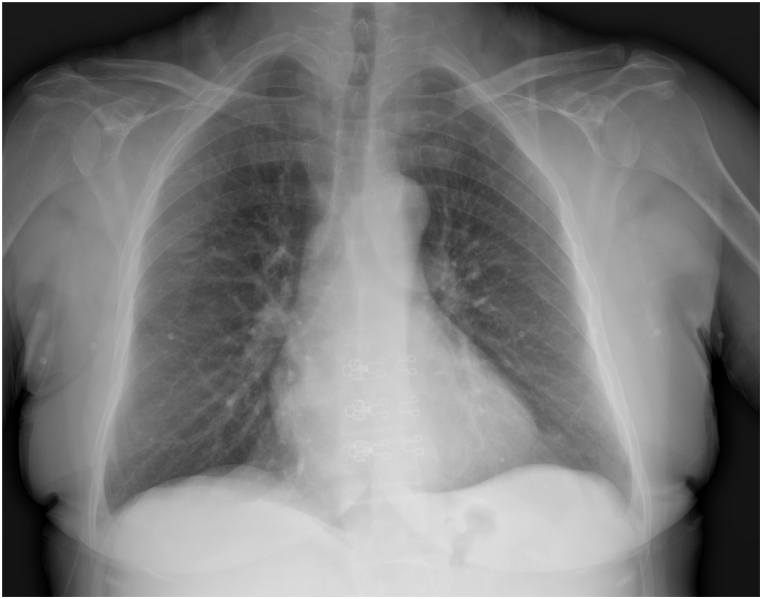
Preoperative chest radiograph demonstrating clear lung fields and absence of pleural effusion.

**Fig. 3:**
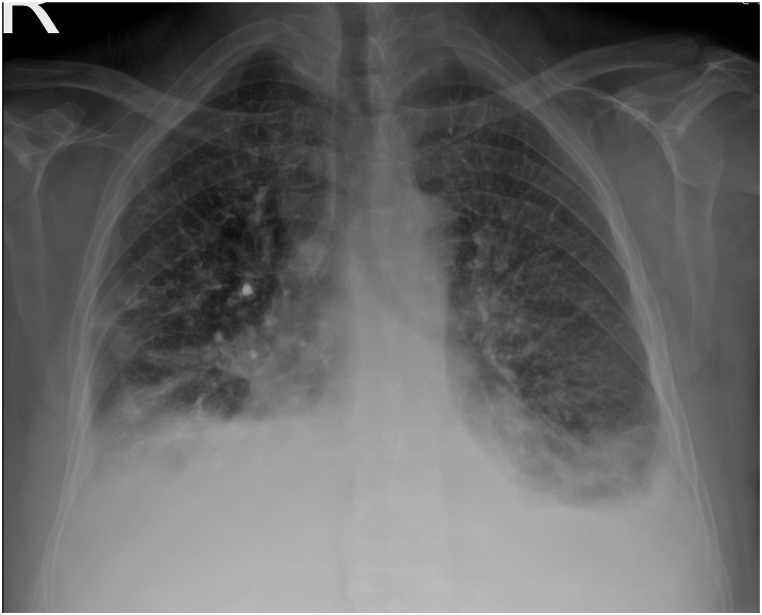
Chest radiograph on postoperative day 7 demonstrating mild reduction in the right pleural effusion and a mild increase on the left side, with bilateral pulmonary opacities.

As inflammatory markers improved (CRP: 80 mg/L; WBC: 9.7 × 10^9^/L) and all microbiological cultures remained negative, all antibiotics were discontinued. On postoperative day 7, chest radiography demonstrated a mild decrease in the right pleural effusion and a mild increase on the left side (Fig. 3). Despite this laboratory improvement, the patient continued to experience respiratory symptoms and significant back pain. A pulmonology consultation raised a high suspicion for pulmonary cement embolism (PCE), prompting non-contrast chest CT scan. Given the persistent back pain, a non-contrast CT scan of the lumbosacral spine was also obtained.

**Fig. 4:**
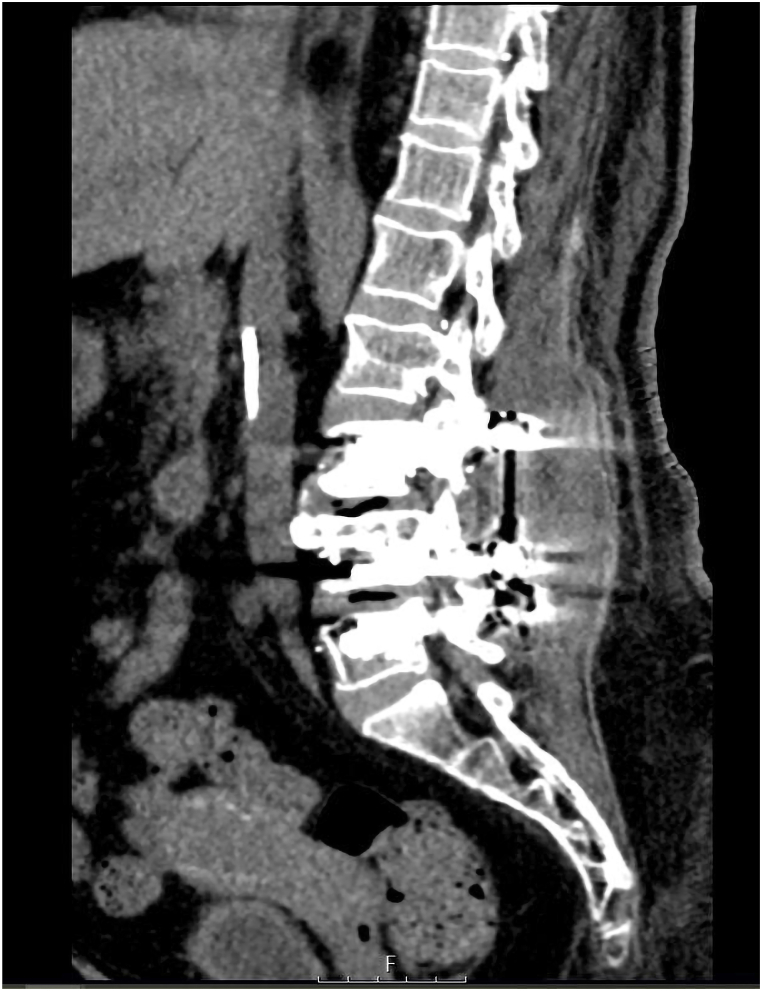
Significant periosteal reaction seen around the screws with osseous calcifications in lines and of significant density, and according to the previous cement surgery these lines are mostly representing thrombosed veins with this cement materials.

**Fig. 5:**
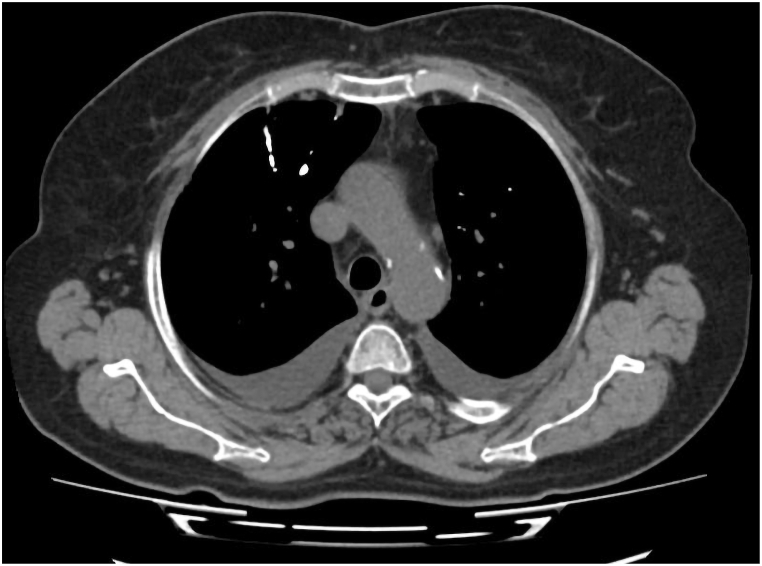
Multiple small tubular branching high dense opacities distributed in the segmental and subsegmental branches of pulmonary arteries bilaterally, predominantly on the right side, representing pulmonary acrylic cement embolism - polymethylmethacrylate (PMMA).

The lumbar CT scan demonstrated the presence of well-positioned pedicle screws at the L3–L5 levels, as well as linear high-density periosteal calcifications that are indicative of thrombosed veins containing PMMA (Fig. 4). The segmental and subsegmental pulmonary arteries were bilaterally characterized by numerous small, branching, high-density opacities, with a preponderance on the right side, as evidenced by the non-contrast chest CT (Fig. 5). Additional discoveries included mosaic attenuation of both lung fields, mild bilateral bronchiectatic alterations, bilateral subpleural atelectatic bands, and patchy subpleural opacities and centrilobular nodules in the right upper and middle lobes. Additionally, bilateral pleural effusions were observed, with a moderate effusion on the right and a modest effusion on the left, as well as an associated fissure effusion. The radiologic findings that had initially raised suspicion for an infectious etiology—right-sided consolidations, centrilobular nodules, and a moderate decrease in the size of the right-sided pleural effusion—were improved in comparison to prior imaging. Nevertheless, a modest increase in the left pleural effusion and a new segmental consolidation in the left lingula were observed.

**Fig. 6:**
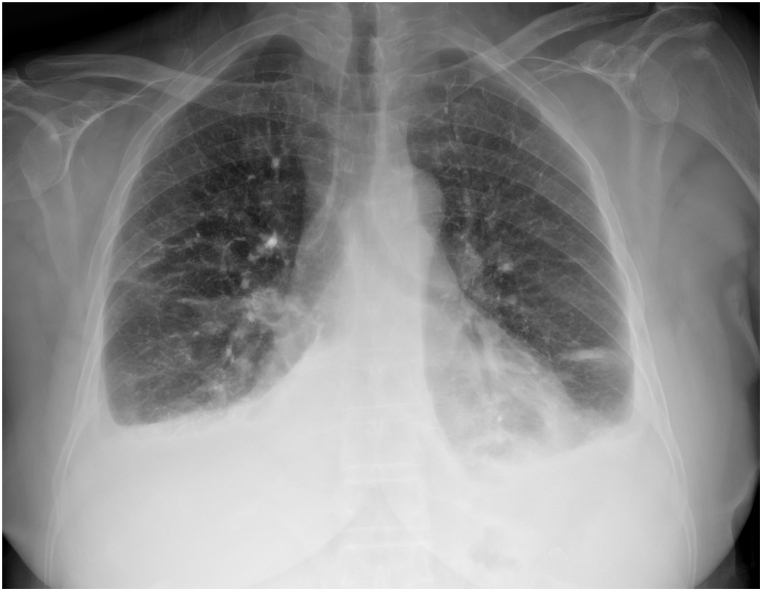
Chest radiograph on hospital day 14 showing interval radiologic improvement, with decreased bilateral opacities and stable pleural effusions.

The patient was treated conservatively with therapeutic enoxaparin (80 mg subcutaneously twice daily) and supplemental oxygen. The discontinuation of oxygen therapy was facilitated by the progressive improvement of her respiratory status and back pain. She had stabilized clinically and radiologically by the fourteenth day of hospitalization, maintaining an oxygen saturation of 92 % on room air and an acceptable chest examination (Fig. 6). Subsequently, she was discharged in satisfactory health. She remained asymptomatic at the two-month follow-up, with an oxygen saturation of 98 % on room air and no back discomfort.

## 3 Discussion

Vertebroplasty is a minimally invasive approach to the treatment of spinal compression fractures. Polymethylmethacrylate (PMMA), also known as bone cement, is injected into the wounded vertebra to provide rapid pain relief and structural reinforcement. Prior to cement injection, a transpedicular needle is inserted under fluoroscopic guidance to access the vertebral body marrow space, a procedure that was initially elucidated by Galibert et al., in 1987. Despite its benefits, vertebroplasty has the potential to cause discomfort, infection, bleeding, and damage to adjacent organs or nerve roots; cement leakage is the primary concern. In this case study, cement pulmonary embolism is highlighted as a rare but potentially hazardous consequence of PMMA vertebroplasty [6,11,12].

Pulmonary cement embolism (PCE) is the consequence of the unintentional extravasation of bone cement into the valveless venous plexus surrounding the vertebra, which enables it to infiltrate the thoracic venous system. Cement initially enters the paravertebral venous plexus, subsequently progresses through the vertebral venous system, and ultimately reaches the pulmonary artery and its branches via the permeable channel. The incidence of PE caused by bone cement ranges from 3 % to 23 %, while PCE has been documented in 2.1 %–26 % of cases. Cement leakage is a frequent occurrence in vertebroplasty surgeries, occurring in 30–75 % of cases, despite its lower prevalence in kyphoplasty (8–33 %). Some of the risk factors that contribute to the development of a fracture include the location of the fracture, the number of segments impacted (more prevalent in thoracic vertebrae), the type of bone degradation (greater risk in tumor-related fractures), the cement volume, mixing ratio, viscosity, and the surgical technique. Ren et al.'s retrospective assessment revealed a significant correlation between the volume of injected PMMA and the complication rate, with an average cement volume of 5.34 mL in cases of leakage. Despite the fact that PCE is frequently asymptomatic, there have been a few confirmed cases with symptoms. The most frequently employed imaging modalities for detection are chest X-rays and CT scans, with a detection rate of 1–6.8 %. In 1999, Padovani et al. reported the first known instance of PCE following spinal cement augmentation, while Luetmer et al. (2011) published the largest case series (n = 23). Raising awareness and improving post-procedure follow-up could enhance early detection and care, thereby reducing the risk of complications [7,[9], [10], [11], [12]].

Symptoms such as dyspnea and chest pain, which typically manifest weeks to months after the procedure, may result from mechanical occlusion of pulmonary vessels caused by pulmonary cement embolism (PCE). Nevertheless, the majority of cases are asymptomatic, with studies suggesting that asymptomatic cement extravasation occurs in up to 75 % of vertebroplasty cases, as documented in a meta-analysis by Lee et al. Although the majority of patients do not demonstrate any discernible clinical symptoms, a small number may develop non-life-threatening symptoms, such as dyspnea, tachypnea, tachycardia, cough, hemoptysis, palpitations, and vertigo. In uncommon instances, fatal outcomes and severe complications, including cyanosis, have been documented. Dyspnea is the most frequently reported symptom in symptomatic cases. Nevertheless, no studies have specifically examined the reasons why certain patients develop symptoms while others remain asymptomatic following PCE, despite these observations [6,9,11,12].

Pulmonary cement embolism (PCE) is distinguished by its unique radiographic appearance on imaging. The cement migration into the pulmonary vasculature is suggested by the dispersed dense opacities that form tubular branches within the lung fields on X-ray. The intraluminal branching pattern with hyperattenuation of the pulmonary vessels is revealed by computed tomography (CT), which provides a more detailed assessment. The detection of PMMA emboli is more effective on non-contrast or portal venous phase studies than on CT pulmonary angiography (CTPA). The high radiodensity of PCE on CT, which exceeds 1000 Hounsfield units (HU), is a critical distinguishing characteristic that differentiates it from the hypodense appearance of thrombotic emboli [6,8,9].

Currently, there are no standardized treatment guidelines, and the management of pulmonary cement embolism (PCE) lacks a well-established, evidence-based gold standard. In general, asymptomatic emboli are routinely managed through close monitoring without intervention. The severity of symptoms and the extent of embolization are the determining factors in the determination of treatment for symptomatic cases. Observation or continuous anticoagulation therapy for 3–6 months may be employed to manage mild or asymptomatic cases, typically involving heparin followed by warfarin. Surgical removal is frequently the preferable method in more severe cases, particularly those that involve hemodynamic instability. Multimodal strategies are frequently implemented to optimize patient outcomes, with additional treatment options including antibiotics, cardiopulmonary bypass, percutaneous thrombus removal, and corticosteroids. [10] [11] [12]

## 4 Conclusion

In summary, it is crucial that surgeons and interventionalists exercise greater caution regarding the rare but potentially hazardous pulmonary cement embolism (PCE) that may result from vertebroplasty. It is imperative to adhere to safe cement preparation and injection protocols, which encompass the use of high-viscosity cement, controlled injection rates, precise needle positioning, real-time fluoroscopic monitoring, and the avoidance of overfilling the vertebral body, in order to prevent complications. Optimal patient selection and meticulous procedural technique are also indispensable. This example underscores the importance of maintaining a high index of suspicion for PCE in patients who experience respiratory symptoms following vertebroplasty, as early detection and treatment are crucial. The majority of cement emboli are asymptomatic; however, those that do exhibit symptoms require prompt detection. Non-contrast CT is the most effective method for this purpose due to the high radiodensity of PMMA. Oxygen support and anticoagulation are frequently viable options for conservative treatment. Early detection may be facilitated by routine chest radiographs that are conducted prior to and following complex or high-risk procedures. In order to enhance patient safety and outcomes through vertebral augmentation, it is essential to implement continuous monitoring, process optimization, and physician education.

## CRediT authorship contribution statement

**Haytham Najjar:** Writing – review & editing, Writing – original draft. **Hind Neiroukh:** Writing – review & editing, Writing – original draft. **Layth J.M. Saada:** Writing – review & editing, Writing – original draft. **Mohamad Thalji:** Writing – original draft, Resources. **Kataeb Doudin:** Writing – review & editing, Supervision.

## Ethical approval

no approval is required.

## Consent

Verbal consent taken from the patient.

## Funding sources

No sources of funding

## Funding

No funding was received for this work.

## Declaration of competing interest

The authors declare that they have no known competing financial interests or personal relationships that could have appeared to influence the work reported in this paper.
